# Phylogenomics resolves the evolutionary chronicle of our squirting closest relatives

**DOI:** 10.1186/s12915-018-0517-4

**Published:** 2018-04-27

**Authors:** Gonzalo Giribet

**Affiliations:** Museum of Comparative Zoology & Department of Organismic and Evolutionary Biology, Harvard University, Cambridge, MA 02138 USA

## Abstract

A recent paper in *BMC Biology* has resolved the family relationships of sea squirts, one of our closest invertebrate relatives, by using a large phylogenomic data set derived from available genomes and newly generated transcriptomes. The work confirms previous ideas that ascidians (the sea squirts) are not monophyletic, as they include some pelagic jelly-like relatives, and proposes a chronogram for a group that has been difficult to resolve due to their accelerated genome evolution.

See research article: https://bmcbiol.biomedcentral.com/articles/10.1186/s12915-018-0499-2

## Commentary

Not all animal groups are equally diverse, appreciated, or studied. For example, a lucky snorkeler in a shallow Caribbean reef would get the impression that sponges (Porifera), cnidarians (Cnidaria), echinoderms (Echinodermata), and ascidians (Tunicata or Urochordata) are the dominant benthic animals, more so than other more speciose animal phyla, such as arthropods (Arthropoda), molluscs (Mollusca), or annelids (Annelida). Of these, ascidians (or sea squirts) are perhaps the lesser known or understood. Ascidians are benthic sessile animals, some are solitary, some aggregate, and some form true colonies, sharing the tunic—one of the defining characters of all Tunicata. Among the colonial species, some are encrusting while others resemble a piece of candy. Their closest relatives are pelagic, again, solitary or colonial jelly-like animals. Furthermore, the ascidian *Ciona robusta* is an important model organism—the closest model organism to vertebrates [1]—and one of the earliest animal genomes ever sequenced, soon followed by another eight tunicate genomes; therefore, unprecedented genomic resources are available for this animal phylum. Yet, unlike all the other abovementioned animal groups that dominate the reefs, no comprehensive modern phylogenetic analyses using other than a handful of genes existed for tunicates until now. This major deficiency in animal phylogenetics has been finally addressed in two papers using phylotranscriptomics, including all major tunicate lineages [2, 3].

But let’s step back for a minute. Tunicate phylogeny has been notoriously difficult to resolve with molecular methods, as many of its members have exceptionally accelerated rates of evolution [4] and display remarkable genome reorganization. Until recently, tunicate molecular phylogenies were inferred using a single nuclear ribosomal RNA marker, or one to a few mitochondrial genes. One study analyzed one nuclear and one mitochondrial gene in combination with morphological characters [5].

Classically, tunicates were divided into three classes, Ascidiacea (the benthic ascidians), Thaliacea (the pelagic salps, pyrosomes, and allies), and Appendicularia or Larvacea (the solitary pelagic tunicates that retain the notochord as adults). An additional group of carnivorous abyssal ascidians were sometimes classified in the class Sorberacea, now known to be related to the molgulid ascidians [6]. The ascidians have been divided into Aplousobranchiata, Stolidobranchiata, and Phlebobranchiata, the latter recognized as probably paraphyletic, and giving rise to the other two groups (see a historical review in [5]). However, all molecular phylogenetic analyses published to date found ascidians to be paraphyletic, with thaliaceans closely related to phlebobranchiates and aplousobranchiates, and sometimes considering stolidobranchiates to be the sister group to appendicularians—although the latter were omitted in many of the molecular studies. Many of these relationships, however, found little support in these datasets; Phlebobranchiata was often non-monophyletic. The amount of molecular data thrown at this interesting phylogenetic question was, however, subpar with current practices.

**Fig. 1.**
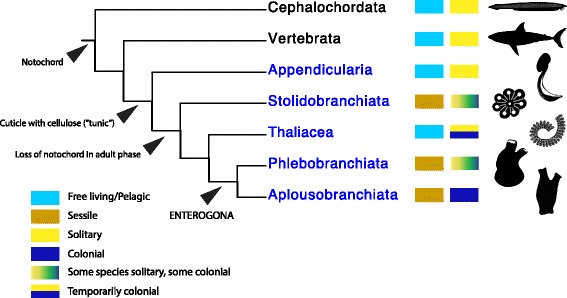
The phylogeny of the tunicate lineages is resolved through phylogenomics. Tunicates are shown in *blue font*. Main characters tracing the diversification of chordates are indicated along the tree. *Light blue squares* indicate free-living chordates (whether benthic or pelagic); *brown* indicates a sessile benthic lifestyle. *Yellow squares* indicate solitary forms; *navy blue* indicates colonial forms, while the *gradient* indicates transitions from solitary to colonial in diverse lineages; the *half-yellow, half-blue square* indicates an alternation of solitary and colonial lifestyle (although some thaliaceans, such as pyrosomes, are permanently colonial). All images from PhyloPics (images credited to B. Duygu Özpolat, Mali’o Kodis, Melissa Frey, Michelle Site, *S. martini*)

Due to their abundance in some environments, to their phylogenetic importance for understanding chordate evolution, and to the considerable available genomic resources [7], the lack of a comprehensive molecular phylogenetic study was surprising. The two new studies both use a partially overlapping set of 18 genomes and transcriptomes [2, 3] and methods designed to ameliorate phylogenenetic error that is pervasive in these large data sets. The results are encouragingly similar—in fact, nearly identical, despite using different orthologue sets and slightly different taxon sampling (Fig. 1). Both studies find maximum support for the main splits within Tunicata, including a sister group relationship of the pelagic Appendicularia to all other tunicates, and a main division between Stolidobranchiata (one of the main clades of ascidians) and the other groups. The other pelagic clade, Thaliacea, appears as the sister group to Phlebobranchiata–Aplousobranchiata. However, the two studies differ in whether Phlebobranchiata, the clade that includes the genetic and developmental model species *Ciona intestinalis*, is monophyletic or paraphyletic with respect to Aplousobranchiata. While these results are not that different from those of the most complete 18S rRNA tree [8], the newer phylogenomic framework provides stronger support for nearly all nodes.

One major gap in our understanding of the evolutionary history of our closest relatives is the lack of a convincing fossil record that could help us understand the early origin and diversification of Tunicata during the Paleozoic. While somehow controversial Vendian tunicates have been proposed [9], it is unlikely that these can help us constrain a molecular tree. Vetulicolians have also been interpreted as tunicates or their closest relatives by some authors due to their bipartite body plan [10], but they have alternatively been assigned to chordate or deuterostome stem groups and, again, would not allow us to perform any internal calibration of the tunicate tree. The new study by Delsuc et al. [2] thus provides the first chronogram—a time tree—for the evolution of this key animal lineage. They use a Bayesian relaxed molecular clock framework and 12 calibration points within vertebrates and echinoderms. Additionally, they set a prior on the root of Deuterostomia, with a mean at the onset of the Cambrian, in an attempt to constrain the floor of the tunicate tree. While this is a starting point, the tunicate chronogram seems to be affected by the extreme evolutionary rates of some of its members when compared to the outgroups, and due to all the calibration points being outside tunicates, the tree results in probable artifacts, as evidenced by the closely related species in the genera *Ciona* and *Molgula* displaying estimated divergences between the Cretaceous and the Jurassic. The authors interpret this result to mean that tunicate “genera” can be very old. I sustain that this may be an artefact due to the lack of internal calibration points, as it is well known that the exclusive use of external calibration groups can have a strong impact on the ingroup’s dates, probably more so in taxa with such divergent local rates as tunicates (see Fig. 1 in [2]). Nonetheless, this study goes beyond what anyone else has been able to do in order to narrate the evolutionary chronicle of ascidians and their closest relatives.

The new study thus provides a comprehensive phylogeny of tunicates, which will allow for a better comparative framework to understand the evolution of genomes between tunicates and the other chordates. It also shows that tunicates divide between those with adult notochords (Appendicularia) and those without, thus contradicting the general idea that appendicularians may be neotenic, given that a notochord is retained in adults of the other two chordate lineages, cephalochordates and vertebrates. The new tunicate phylogeny is thus the first step towards understanding the evolution of morphology in an animal group that has made multiple transitions to coloniality (sometimes with sexual and asexual life cycles), has switched between the benthos and the pelagos at least twice, and has colonized the deep sea on multiple occasions, where species have evolved macrophagy or “carnivory” independently.

## References

[1] Delsuc F, Brinkmann H, Chourrout D, Philippe H (2006). Tunicates and not cephalochordates are the closest living relatives of vertebrates. Nature.

[2] Delsuc F, Philippe H, Tsagkogeorga G, Simion P, Tilak M-K, Turon X, López-Legentil S, Piette J, Lemaire P, Douzery EJP. A phylogenomic framework and timescale for comparative studies of tunicates. BMC Biol. 2018;16:39. 10.1186/s12915-018-0499-2 PMC5899321 29653534

[3] Kocot KM, Tassia MG, Halanych KM, Swalla BJ (2018). Phylogenomics offers resolution of major tunicate relationships. Mol Phylogenet Evol.

[4] Tsagkogeorga G, Turon X, Galtier N, Douzery EJP, Delsuc F (2010). Accelerated evolutionary rate of housekeeping genes in tunicates. J Mol Evol.

[5] Stach T, Turbeville JM (2002). Phylogeny of Tunicata inferred from molecular and morphological characters. Mol Phylogenet Evol.

[6] Tatián M, Lagger C, Demarchi M, Mattoni C (2011). Molecular phylogeny endorses the relationship between carnivorous and filter-feeding tunicates (Tunicata, Ascidiacea). Zool Scr.

[7] Lemaire P, Piette J (2015). Tunicates: exploring the sea shores and roaming the open ocean. A tribute to Thomas Huxley. Open Biol.

[8] Govindarajan AF, Bucklin A, Madin LP (2011). A molecular phylogeny of the Thaliacea. J Plankton Res.

[9] Fedonkin MA, Vickers-Rich P, Swalla BJ, Trusler P, Hall M (2012). A new metazoan from the Vendian of the White Sea, Russia, with possible affinities to the ascidians. Paleontol J.

[10] Lacalli TC (2002). Vetulicolians–are they deuterostomes? Chordates?. BioEssays.

